# A new technique of anterograde puncture for chronic limb-threatening ischemia with superficial femoral artery flush occlusion: vascular sheath fenestration

**DOI:** 10.1007/s00380-025-02534-6

**Published:** 2025-03-14

**Authors:** Zhaopeng He, Boyu Wang, Haoyong Meng, Lei Zhang, Qingfu Zhang

**Affiliations:** 1 Department of Vascular Surgery, Hebei Key Laboratory of Colorectal Cancer Precision Diagnosis and Treatment, The First Hospital of Hebei Medical University, No. 89 Donggang Rd, Shijiazhuang, 050000 Hebei China; 2 Department of Intervention, The First Hospital of Hebei Medical University, Shijiazhuang, China; 3 Department of Burn and Wound Repair Center, Hebei Medical University Third Hospital, No. 139 Ziqiang Rd, Shijiazhuang, 050000 Hebei China

**Keywords:** Chronic limb-threatening ischemia, Superficial femoral artery flush occlusion, Vascular sheath fenestration, Anterograde puncture, Global Limb Anatomic Staging System, Vascular sheath fenestration assisted anterograde puncture technique for superficial femoral artery flush occlusion

## Abstract

We introduce a technique for treating chronic limb-threatening ischemia with superficial femoral artery flush occlusion, facilitating intravascular treatment when conventional anterograde puncture is challenging. This retrospective study reviewed 37 patients who underwent vascular sheath fenestration assisted anterograde puncture to complete endovascular treatment for chronic limb-threatening ischemia from December 2022 to December 2023. All patients had superficial femoral artery flush occlusion, meeting chronic limb-threatening ischemia diagnostic criteria. Evaluations included intraoperative radiation dose, technical success rate, patency rate, limb retention rate, and postoperative complications from surgery to a 12-month follow-up. The mean age of the patients was 70 ± 10 years, with an age range of 46 to 90 years. A significant proportion of the cases presented with severe chronic limb-threatening ischemia, with 78.4% classified as Rutherford ≥ 5, 51.3% as WiFi ≥ 3, and 97.3% as Global Limb Anatomic Staging System III. In all surgical procedures, a plain old balloon angioplasty was utilized for anterograde dilation. Subsequently, based on angiographic findings, treatment involved either drug-coated balloon dilation combined with stent implantation or drug-coated balloon dilation alone. Successful revascularization was achieved in all cases, resulting in marked clinical and hemodynamic improvements, as evidenced by the mean ankle-brachial index increasing from 0.49 preoperatively to 0.86 postoperatively. The 12-month follow-up outcomes were as follows: limb salvage rate of 94.6%, primary patency rate of 83.8%, assisted primary patency rate of 91.9%, and secondary patency rate of 94.6%. The incidence of postoperative complications was 8.1%. The average duration of hospital stay was 8.43 ± 2.72 days. The vascular sheath fenestration assisted anterograde puncture technique demonstrates favorable surgical outcomes and merits consideration as a viable treatment option for chronic limb-threatening ischemia patients with superficial femoral artery occlusion.

## Introduction

For advanced GLASS (Global Limb Anatomic Staging System) and WIfI (Wound, Ischemia, and foot Infection) CLTI patients with highly complex anatomic lesions, bypass surgery is the preferred treatment. This recommendation is primarily due to the presence of multi-segmental vascular disease, intricate anatomic considerations, and its higher long-term patency rate compare with endovascular treatment (EVT) [1, 2]. Conversely, these patients are typically not ideal candidates for the significant risks associated with bypass surgery. The perioperative mortality and complication rates associated with bypass surgery are notably high. Moreover, the use of bypass surgery is currently limited in patients with suboptimal distal outflow tract conditions. However, EVT offers the benefits of reduced trauma and quicker recovery, which can also effectively enhance blood flow and alleviate symptoms [3].

The EVT of flush superficial femoral artery (SFA) occlusion remains a contentious issue within the medical community, as it complicates the direct placement of the vascular sheath in the superficial artery, and antegrade puncture may result in an unsafe sheath location. Previously, this was considered a contraindication for most intravascular inguinal interventions. However, contemporary practice involves a thorough analysis of the patient’s specific circumstances to determine the most appropriate surgical approach. The EVT of SFA flush occlusion typically necessitates a specialized puncture approach. Currently, the predominant techniques include the contralateral cross-over approach and ipsilateral retrograde puncture of the popliteal or anterior tibial artery. However, the former is challenging in cases of abnormal iliac artery anatomy or previous iliac artery interventions. The latter approach requires advanced puncture skills from the surgeon and carries risks such as CFA dissection and deep femoral artery (DFA) occlusion. In addition, the retrograde method is not feasible when distal artery puncture is impeded by stenosis, occlusion, or patient discomfort during EVT. Both approaches present challenges in the management of subknee artery disease.

Consequently, this paper introduces a novel puncture approach strategy, termed the vascular sheath fenestration (VSF) assisted anterograde puncture technique. This approach enhances operational stability and safety, facilitates the navigation of the guide wire into the superficial artery opening, and provides robust support for the guide wire and catheter, and it facilitates the treatment of diseases associated with DFA.

## Materials and methods

### Study design

We conducted a retrospective review of 37 cases involving patients who underwent VSF procedures at a single center between December 2022 and December 2023. Informed consent was obtained from all participants, and they were scheduled follow-up visits for a minimum duration of 12 months. This study was conducted in accordance with the Declaration of Helsinki.

*Inclusion criteria*: We enrolled patients with SFA flush occlusion as criteria for angiography. And the clinical inclusion criteria for these lesions were lifestyle-limiting critical limb-threatening ischemia (CLTI), corresponding to Rutherford grades 4, 5, and 6.

*Exclusion criteria*: Patients with underlying causes of lower limb ischemia, such as acute thromboembolism, trauma, or autoimmune diseases; those with CFA lesions; and individuals with contraindications to intravascular procedures, including allergies to contrast agents.

*Study end points*: We have analyzed intraoperative radiation exposure and the efficacy of the procedure 12 months post-surgery. Primary endpoints such as technical success rate and patency rate were included, while secondary endpoints included limb retention rate and postoperative complications.

### Study protocol

Preprocedural work-up and evaluation. We retrospectively analyzed demographic data, body mass index, current and historical medical information, treatment indications, preoperative computed tomography arteriography (CTA), intraoperative and postoperative complications, and follow-up Doppler ultrasound (ABI) records. Imaging assessments revealed arteriosclerosis and occlusion in the lower extremities, accompanied by symptoms of lower extremity arterial ischemia, thus meeting the diagnostic criteria of CLTI [4]. Preoperative CTA and intraoperative digital subtraction angiography (DSA) were employed to guide the selection of surgical techniques, as well as balloon and stent choices. Clinical and limb threat stages were assessed using the Rutherford classification, GLASS, and the wound, ischemia, and foot infection (WIfi) grading system.

The study adhered to the Society for Vascular Surgery (SVS) guidelines for defining and reporting outcomes related to technical success, primary, assisted, and secondary patency, as well as stenosis/restenosis. One month following surgery, the patient underwent an Ankle-Brachial Index (ABI) assessment at the vascular surgery clinic, with subsequent outpatient evaluations scheduled every 3 months. All examinations were conducted independently by a consistent vascular technician within the vascular surgery examination room and were subsequently reviewed by the vascular surgery outpatient physician.

Data analysis was performed utilizing SPSS version 26.0 software (Chicago, IL, USA). Continuous variables are presented as mean ± standard deviation or quartiles, while categorical variables are expressed as frequencies and percentages.

**Fig. 1:**
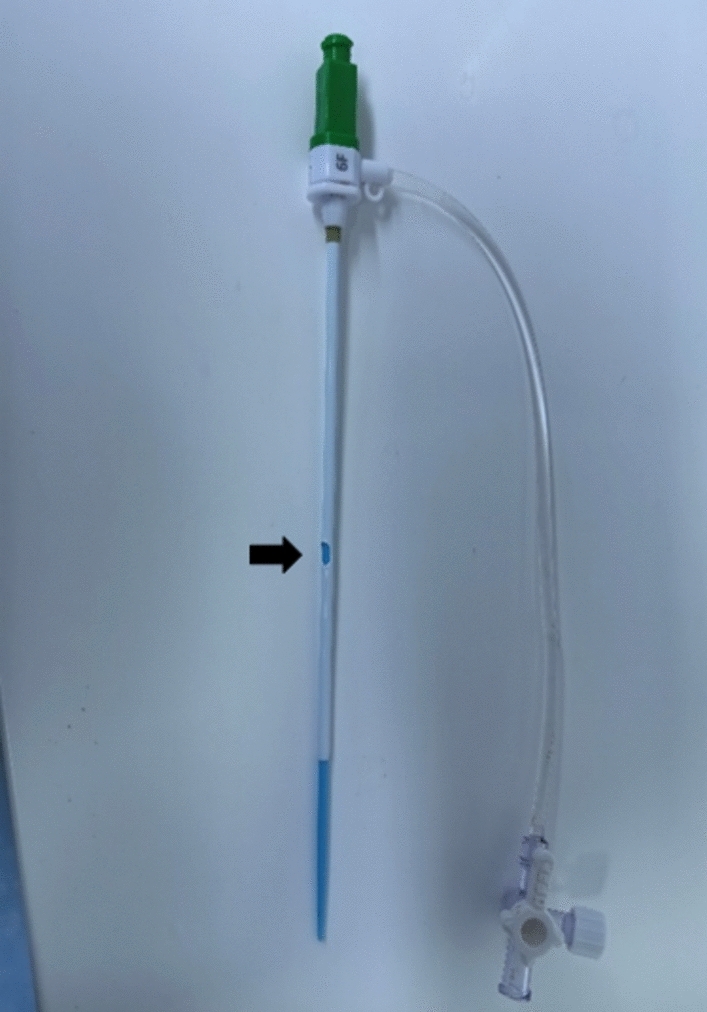
The figure shows an example of vascular sheath fenestration, and the arrow points to the fenestration

**Fig. 2:**
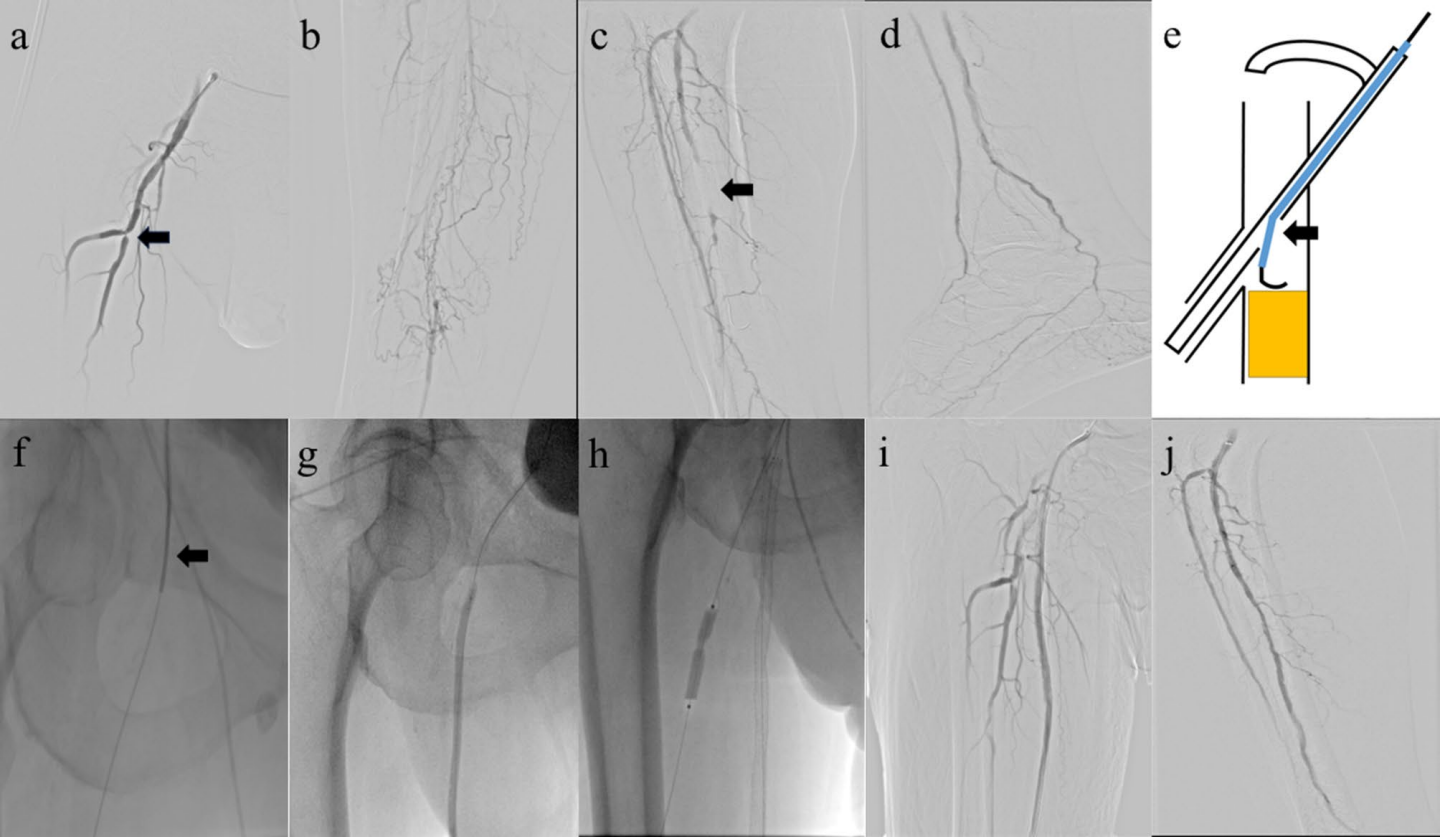
Procedure images: **a** The angiography showed that the SFA flush occlusion, the vascular sheath was placed in the DFA, and the DFA indicated by the arrow was severely narrow. **b** Long superficial femoral artery occlusion. **c** Angiography showed segmental occlusion of the anterior tibial artery and posterior tibial artery. **d** The angiography of the inferior malleolar artery was well. **e** As shown in the schematic, the arrow points to the fenestration of the vascular sheath, which is placed in the deep femoral artery at this time. Adjust the direction of the fenestration according to the position of the sheath side arm and preoperative CTA so that it is directly opposite the opening of the superficial femoral artery. **f** The catheter followed by the guide wire selected the SFA. **g** Used a balloon to dilate the SFA. **h** A stent was placed in the SFA, and a balloon was used to dilate the DFA stenosis. **i** The SFA and DFA were well showed after treatment. **j** The anterior tibial artery and posterior tibial artery showed well after treatment

*Endovascular procedures*: All procedures were performed under local anesthesia, with the puncture site infiltrated with 2% lidocaine. In a supine position, the patient utilized a micro-puncture system to puncture the CFA against the femoral head, ensuring that the skin puncture point was slightly higher than the artery puncture point to facilitate the tunneling of the sheath under the skin. The needle was inserted into the middle and upper segment of the CFA. Following a successful puncture, its position in the CFA is confirmed via angiography and the guide wire was then advanced into the DFA. The 6F Termel vascular sheath was chosen, and a central point was identified on the sheath wall opposite the side arm opening to facilitate a circular fenestration incision using a scalpel, with a diameter of approximately 2 mm (Fig. 1). The selection of a 2 mm diameter is justified by the fact that the sheath itself measures approximately 2 mm in diameter. This dimension ensures that any instrument passing through the sheath can also traverse the window, while exceeding 2 mm in diameter heightens the risk of sheath rupture. The vascular sheath was then advanced along the guide wire. The position of the fenestration was adjusted to align with the openning of the SFA under preoperative CTA guidance, based on the location of the sheath’s side arm. A VER catheter and a 1.5-m hydrophilic-coated guide wire were utilized through the window to access the superficial artery opening and address the occluded vascular segment. At this stage, the VSF was completed. In cases the SFA orifice is difficult to visualize or the subintimal space is entered, the retrograde puncture technique can be used to place the guidewire retrogradely through the distal outflow tract (femoro-popliteal or infrapopliteal artery) to the SFA opening, providing precise positioning for the catheter and guide wire selected for passage through the fenestration and re-enter the true lumen. Subsequent treatment is tailored to the patient’s specific lesions, potentially involving a drug-coated balloon with or without stent placement (Fig. 2).

*Postprocedural.* Aspirin plus clopidogrel (DAPT) is frequently employed for 6 months after interventions. Postoperative follow-up assessments were conducted at 1, 3, 6, and 12 months, or when patients reported a recurrence of symptoms or clinical signs. The clinical evaluation encompassed the assessment of improvements in symptoms of resting pain, alterations in the ABI, ulcer wound healing, and limb preservation. Vascular patency or the presence of restenosis/occlusion was evaluated based on clinical symptoms and ABI measurements. In cases where clinical symptoms and ABI data indicated potential restenosis or re-occlusion, a computed tomography angiography (CTA) was performed to further assess the patient’s condition.

### Definitions

*Flush occlusion.* Flush occlusion is a chronic occlusion that starts at or less than 1 cm distal to the origin of the artery by visual estimation [5].

*Technical success*: Restoration of continuous pulsatile blood flow from the groin to the foot was achieved, with residual stenosis maintained at less than 30% throughout.

*Clinical success*: Clinical symptoms were either alleviated or resolved completely.

Primary patency. No instances of stenosis exceeding 30% or occlusion were observed in the target vessels.

*Assisted primary patency*: In cases where the target vessel was not occluded, surgical intervention was undertaken to enhance blood-flow patency.

*Secondary patency*: When target vessel occlusion occurred, surgical intervention was performed to re-establish blood-flow patency.

## Results

The study sample comprised 33 male and 4 female participants, with ages ranging from 46 to 90 years (mean age: 70 years). Within this cohort, 20 out of 37 patients (54.1%) were identified as smokers, 32 out of 37 patients (86.5%) presented with underlying medical conditions, there are 21 out of 37 patients (56.8%) with diabetes, 26 out of 37 patients (70.3%) with hypertension, 4 out of 37 patients (10.8%) with dyslipidemia, 10 out of 37 patients (27.0%) with cardiovascular diseases, and 11 out of 37 patients (29.7%) with cerebrovascular diseases. In addition, 3 out of 37 patients (8.1%) required hemodialysis, and all participants underwent ABI examination prior to surgery. During statistical analysis, the ABI for patients with uncompressed arteries was recorded as 0. The demographic and clinical characteristics are detailed in Table 1.

**Table 1:** The demographic and clinical characteristics^a^

Variables	
Age—years	70 ± 10
	46–90
BMI obesity	1 (2.7%)
Male sex	33 (89.2%)
Smoking	20 (54.1%)
Diabetes	21 (56.8%)
Hypertension	26 (70.3%)
Hyperlipemia	4 (10.8%)
CAD	10 (27.0%)
Cerebrovascular disease	11 (29.7%)
COPD	1 (2.7%)
Hemodyalisis	3 (8.1%)
Preoperative ABI	0.52 (0.33, 0.66)

BMI: Body mass index; CAD: Coronary Artery Disease; COPD: Chronic Obstructive Pulmonary Disease; ABI: Ankle-Brachial Index

^a^Continuous data are presented as the mean ± standard deviation or quartile; categorical data are given as the count (percentage)

All treated patients exhibited varying degrees of occlusion at the flush of the SFA. 28 patients (75.7%) presented with subpatellar artery lesions necessitating surgical intervention. Notably, 36 out of 37 cases (97.3%) presented with highly complex subinguinal lesions classified as stage GLASS III, with only one case categorized as moderately complex (stage GLASS II). Among the cohort, 29 patients exhibited severe symptoms, including gangrene or ulceration classified as Rutherford grade 5 or higher, representing 78.4% of the total population. In addition, 19 patients were identified with a WIFI grade of 3 or above, constituting 51.3% of the total. The characteristics of the arterial lesions are presented in Table 2.

**Table 2:** The characteristics of the arterial lesions

Lesion characteristics	
GLASS stage	
2	1 (2.7%)
3	36 (97.3%)
FP	
2	9 (24.3%)
3	17 (45.9%)
4	11 (29.7%)
IP	
0	
1	
2	
3	18 (48.6%)
4	19 (51.4%)
IM	
P0	19 (51.4%)
P1	18 (48.6%)
P2	0 (0)
Rutherford category	
4	8 (21.6%)
5	8 (21.6%)
6	21 (56.8%)
WIfI stage	
2	18 (48.6%)
3	4 (10.8%)
4	15 (40.5%)

GLASS: Global Limb Anatomic Staging System; FP: Femoropopliteal; IP: infrapopliteal; IM: Infra-malleolar; WifI: Wound: Ischemia: foot Infection

The intraoperative data collected included a puncture time of 2.33 ± 0.85 min, a radiation dose of 510.69 ± 372.60 mGy, a dose area product (DAP) of 8783.61 ± 4903.19 µGy m^2^, and a fluoroscopy time of 20.58 ± 8.71 min. The contrast volume used was 56 ± 34 mL, and the total procedure duration was 109.11 ± 26.31 min. Postoperative outcomes indicated an average hospital stay of 8.43 ± 2.72 days. Among the 37 patients, there was one case of postoperative inguinal hematoma (He was successfully treated with conservative management), one case of postoperative pneumonia (He was successfully treated with conservative management), and one patient experienced other complications (in-stent thrombosis during the perioperative period, which was managed with mechanical thrombus removal). The specific intraoperative treatment strategies, including the use of balloons and stents, were determined collaboratively by three doctors with titles of deputy director and above from the vascular surgery department, based on preoperative assessments and intraoperative angiographic findings. Routine procedures involved the application of plain old balloon angioplasty (POBA) for the femoro-popliteal lesions and below knee lesions. In cases where angiography indicated residual stenosis greater than 30% or the presence of flow-limiting dissection, drug-coated balloon dilation combined with stent implantation was performed; otherwise, drug-coated balloon dilation was administered independently. Detailed intraoperative data and postoperative complications are presented in Table 3.

**Table 3:** Intraoperative data and postoperative complications

Procedure characteristics	
Puncture time, min	2.33 ± 0.85
Radiation	
Ray dose, mGy	510.69 ± 372.60
DAP, uGym^2^	8783.61 ± 4903.19
Fluoroscopy time, min	20.58 ± 8.71
Contrast, ml	56 ± 34
Procedure duration, min	109.11 ± 26.31
Hospital stays, day	8.43 ± 2.72
Complication	
Hematoma in the groin	1 (2.7%)
Pseudoaneurysm	0 (0)
MI	0 (0)
Pneumonia	1 (2.7%)
Other complications	1 (2.7%)
Treatment method	
Femoropopliteal lesions	
Drug-coated balloon	37 (100%)
Bare metal stent	18 (48.6%)
Below knee lesions	
Non	9 (24.3%)
Drug-coated balloon	28 (75.6%)
Stent	6 (16.2%)

DAP: cumulative dose area product; MI: myocardial infarction

**Fig. 3:**
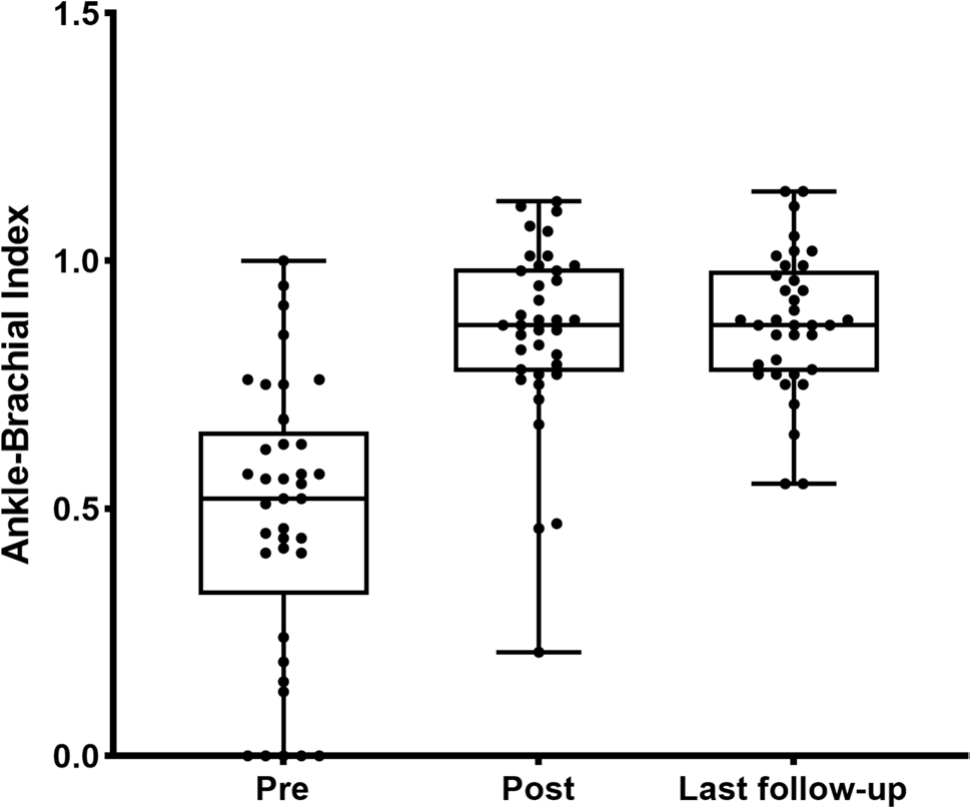
The box-plot shows the changes in the patient’s preoperative ABI, pre-discharge ABI, and postoperative follow-up ABI, with an overall upward trend

**Fig. 4:**
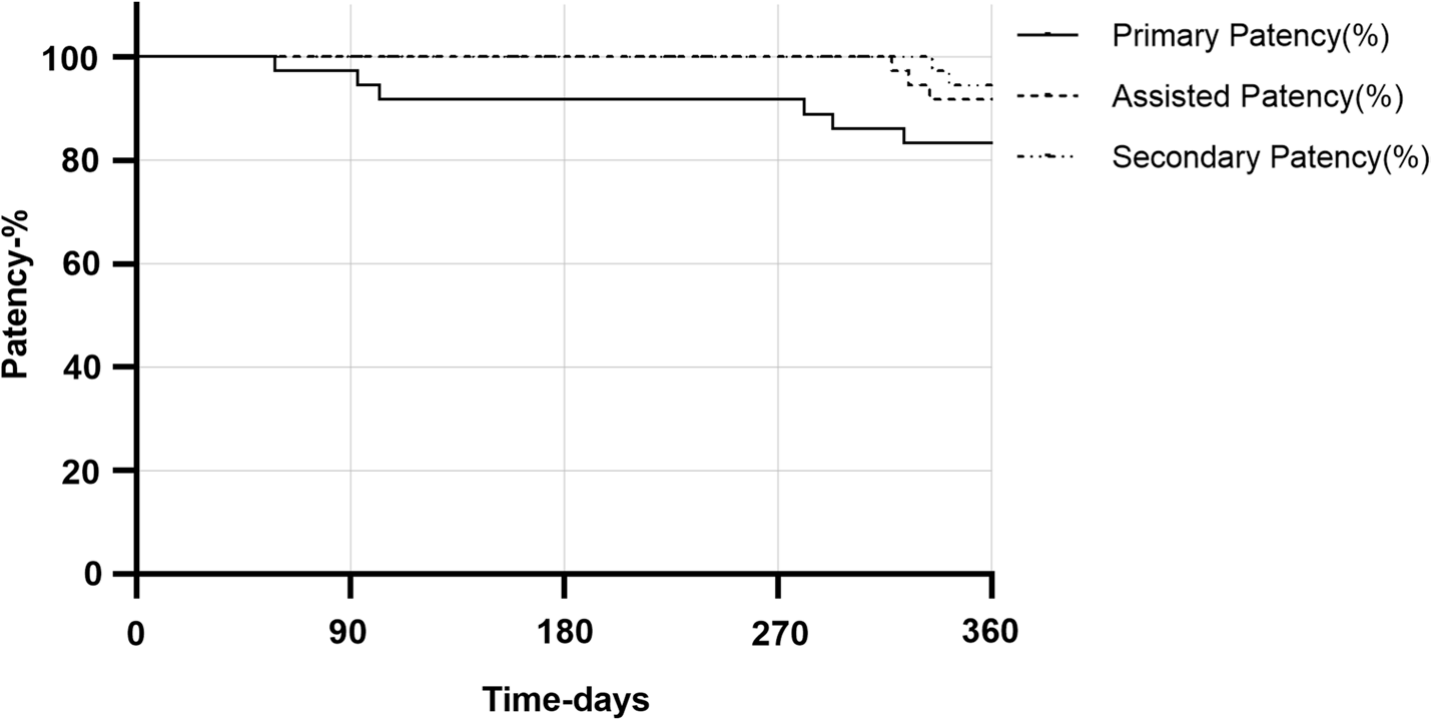
Patency (primary, assisted, secondary) represented by Kaplan–Meier survival curves

Among the cohort of 37 patients, 2 patients presented challenges in selecting a shallow artery even after employing the VSF. For these cases, retrograde puncture of the anterior tibial artery or the popliteal artery was utilized to address lesions at the opening of the SFA and to continue treatment of subknee artery lesions. The remaining patients experienced technical success, with all cases achieving both clinical and hemodynamic success. The ABI showed significant improvement, increasing from 0.49 ± 0.28 preoperatively to 0.86 ± 0.19 postoperatively (Fig. 3). At the 12-month follow-up, the VSF technique demonstrated a primary patency rate of 83.8%, an assisted patency rate of 91.9%, and a secondary patency rate of 94.6% (Fig. 4). During the follow-up period, major amputation was performed in 2 out of 37 cases (5.4%). In these two patients, foot gangrene had developed prior to surgery, and infection spread following revascularization, necessitating subknee amputation. No other major adverse events were reported, and all patients exhibited fewer symptoms compared to their preoperative state. The wound healing process was satisfactory.

## Discussion

In the management of CLTI, reestablishing direct blood flow to the foot is crucial for achieving effective treatment outcomes. Although the long-term outcomes of bypass surgery are generally regarded as superior to those of EVT, bypass surgery is associated with greater risk due to its invasive nature and the requirement for general anesthesia, leading to a higher complication rate compared to EVT. EVT offers several advantages, including a significant reduction in surgical trauma, decreased patient recovery time, and enhanced overall treatment efficacy. Compared to traditional open surgery, EVT is a less invasive approach, making it particularly suitable for high-risk patients. Minimally invasive techniques have advanced in recent years, and it is still controversial whether bypass surgery is feasible for patients with poor distal outflow tract condition [6]. Consequently, vascular surgeons are increasingly favoring EVT as the preferred treatment option. In this study, 32 out of 37 patients (86.5%) presented with various underlying conditions, and the average patient age was relatively advanced (70 ± 10 years), making EVT a more suitable treatment choice.

In cases where there is occlusion at the opening of the SFA, the direct placement of a vascular sheath into the superficial artery via ipsilateral groin femoral artery puncture is not feasible. Thus, selecting the most appropriate approach has become a focal point of clinical consideration. Consequently, various specialized puncture techniques have been developed to ensure the stability of the vascular sheath and the surgical procedure. Two commonly used types include the contralateral cross-over approach and ipsilateral retrograde puncture of the popliteal or anterior tibial artery. For patients with subpatellar artery disease, both techniques present management challenges. The first method is limited by its inability to access the subpatellar artery, often resulting in incomplete or ineffective revascularization. A meta-analysis reported that the primary patency rate and amputation rate for patients with CLTI treated with this approach were 62% and 20% at 1 year, respectively. The second method fails to provide sufficient support, thereby complicating the management of subpatellar arteries. And in the case of this approach, the force is mechanically transmitted outward (i.e., toward the DFA), which is thought to reduce the success rate of CTOs. In this study, the majority of patients were classified as GLASS Stage III, with 28 patients (75.7%) presenting with subknee artery lesions necessitating surgical intervention. The choice of anterograde puncture kept advantageous for the treatment of these lesions. Consequently, when feasible, ipsilateral anterograde puncture remains the preferred approach.

Some researchers have introduced the technique of double guide wire strand deep anchoring, which aims to stabilize the vascular sheath by inserting a guide wire into the DFA [7]. Despite offering some degree of stability, this method reduces the length of the vascular sheath within the vessel and carries the risk of sheath prolapse. In addition, the risk of sheath prolapse and the insertion of a double guide wire into the vascular sheath may increase the risk of bleeding. A case report suggested that a customized curved micro-puncture needle could be employed to directly advance the puncture needle from the CFA to the occluded SFA under ultrasound guidance for therapeutic purposes [8]. While this approach facilitates the establishment of a pathway through the SFA, the VSF technique is superior in terms of operational simplicity and safety.

This technique offers a distinct advantage in managing the SFA. With the VSF procedure, it is possible to wire straight into the SFA mechanically. And one notable benefit of the VSF technique, as utilized in this study, is the ability to insert the entire vascular sheath into the blood vessel. Furthermore, the presence of a catheter and guide wire at the fenestration site minimizes the likelihood of vascular sheath withdrawal, thereby maximizing the stability of the vascular sheath, ensuring operational safety, and preventing bleeding events associated with sheath withdrawal. Furthermore, the operational procedure of this method is straightforward, enabling junior doctors at our center to successfully perform punctures shortly after familiarizing themselves with the technique. The brief learning curve facilitates widespread adoption. In comparison to the contralateral femoral artery puncture cross approach, this method offers robust support for treating subknee artery lesions and can concurrently address DFA lesions when present. Of the 37 patients in this study, this approach achieved a 100% rate of hemodynamic success at the end of the surgery, demonstrated a favorable 12-month patency rate (Fig. 4), and resulted in a significantly reduced amputation rate of 2.7% (1/37) in cases of high complexity endovascular therapy (EVT).

The study is limited by its small sample size and lack of a randomized controlled design. Further research is warranted to provide more robust evidence.

## Conclusion

For patients with chronic limb-threatening ischemia (CLTI) and SFA flush occlusion, the VSF technique is a safe and effective option for establishing vascular access, particularly when concurrent treatment of infrapopliteal disease is required.

## Data Availability

The data that support the findings of this study are available from the corresponding author, upon reasonable request.
